# Medical Provident Institution of Scotland

**Published:** 1831

**Authors:** 


Medical Provident Institution of Scotland.

In our Review for March, 1830, we called the attention of our readers to this excellent institution, and to that Number we refer them for a detailed account of its objects and advantages. Since then the Institution has held its fourth annual general meeting, and it gives us pleasure to learn from the Report of the Directors which was then read, that it is becoming better known, its objects better understood, and in consequence that it is rising in favour with the profession. The Directors having recommended that the benefits of the Institution should be thrown open to the families and relations of the members of the medical profession, this was unanimously approved of by the meeting. We are inclined to think that this will render the Institution more popular. Members of the profession will now have it in their power to make provision in it against the casualties and contingencies of life, not only for themselves, but also for those in whom they are most deeply interested.

We may remind our readers, that, the principle of this society is that of mutual assurance. Its whole funds arise from the contributions of the members, and belong entirely to them, there being no body of proprietors. "Every five years the affairs of the Institution shall be brought to a balance, and two-thirds of the surplus divided among the contributors, and the remaining one-third carried forward as a guarantee, and to meet any extraordinary contingencies."

